# Hepcidin from the Chinese Spiny Frog (*Quasipaa spinosa*) Integrates Membrane-Disruptive Antibacterial Activity with Macrophage-Mediated Protection Against *Elizabethkingia miricola*

**DOI:** 10.3390/genes16121450

**Published:** 2025-12-04

**Authors:** Fen Qiao, Xin-Yi Qian, Yi-Kai Feng, Jie Chen

**Affiliations:** 1 College of Agriculture and Biotechnology, Lishui University, Lishui 323000, China; qiaofen121@163.com (F.Q.); lsxyqianxinyi@126.com (X.-Y.Q.); lsufyikai@126.com (Y.-K.F.); 2 Industrial College of Traditional Chinese Medicine and Health, Lishui University, Lishui 323000, China; 3 College of Animal Sciences, Zhejiang University, Hangzhou 310058, China

**Keywords:** hepcidin, Chinese spiny frog, primary macrophage, *Elizabethkingia miricola*, aquaculture disease control

## Abstract

**Background/Objectives**: Hepcidin is a cysteine-rich antimicrobial peptide that links iron homeostasis and innate immunity in vertebrates, but its functions in amphibians remain poorly understood. The Chinese spiny frog (*Quasipaa spinosa*) is an economically important species that suffers serious losses from bacterial diseases. This study aimed to identify and functionally characterize a hepcidin homolog (QsHep) from *Q. spinosa*, focusing on its antibacterial activity, immunomodulatory effects on primary macrophages, and protective efficacy against *Elizabethkingia miricola* infection. **Methods**: The *QsHep* gene was cloned and analyzed, its tissue distribution and inducible expression were examined by qRT-PCR, and the synthetic peptide was tested for antimicrobial, membrane-disruptive, and immunomodulatory activities in vitro, as well as for in vivo protection in an *E. miricola* infection model. **Results**: *QsHep* encodes a typical preprohepcidin with a signal peptide, prodomain, and a conserved mature peptide containing eight cysteine residues. *QsHep* was widely expressed, with the highest levels in liver, and was significantly upregulated in liver and spleen following bacterial challenge. Synthetic QsHep displayed broad-spectrum antibacterial activity, including strong inhibition of *E. miricola*, and induced dose-dependent membrane damage in *E. miricola*. QsHep showed no obvious cytotoxicity but significantly enhanced chemotaxis, phagocytic activity, and respiratory burst in primary macrophages. In vivo, QsHep treatment markedly improved the survival of *E. miricola*-infected frogs in a dose-dependent manner. **Conclusions**: QsHep is an amphibian hepcidin that combines membrane-disruptive antibacterial activity with the activation of macrophage effector functions and confers significant protection against bacterial infection in vivo. These findings expand our understanding of hepcidin-mediated innate immunity in amphibians and highlight QsHep as a promising peptide candidate for controlling bacterial diseases in frog aquaculture.

## 1. Introduction

Antimicrobial peptides (AMPs) are a diverse class of small peptides, typically 12–50 amino acids in length, that are widely distributed across bacteria, plants, invertebrates, and vertebrates [1,2,3,4]. As key effectors of the innate immune system, AMPs constitute a first line of defense against invading pathogens [5,6]. Most AMPs are short, cationic, and amphipathic, displaying considerable sequence diversity, and act against a broad spectrum of bacteria, fungi, and viruses [7,8,9,10]. In addition to their potent antimicrobial activity, many AMPs exhibit favorable properties such as low toxicity to eukaryotic cells, good solubility, high thermal stability, low molecular weight, and a relatively low propensity to induce resistance, making them attractive templates for anti-infective drug development [11].

Hepcidin is a cysteine-rich AMP initially purified from human blood ultrafiltrate and urine [12,13]. Since their discovery, hepcidins have been identified in various vertebrate lineages, including fish and reptiles [14,15,16,17], and are characterized by a highly conserved, disulfide-stabilized β-sheet structure containing 6–8 cysteine residues, which are essential for their antimicrobial activity [13,18,19]. Hepcidin exhibits bactericidal activity against a range of pathogens, such as *Escherichia coli*, *Pseudomonas aeruginosa*, *Enterococcus faecium*, and *Staphylococcus epidermidis* [13,18,19]. It was later recognized as a central hormone in iron homeostasis, down-regulating the iron exporter ferroportin 1 (FPN1) through binding, internalization, and degradation [20,21,22]. Mammalian hepcidins also possess antifungal and antimalarial activities, for instance against *Plasmodium berghei* [13,23,24], highlighting their dual role at the interface of metal metabolism and host defense.

In amphibians, however, knowledge of hepcidin remains very limited. To date, the *hepcidin* gene has been reported only in *Xenopus tropicalis*, where molecular characterization and expression patterns have been described [25], but the antimicrobial and immunomodulatory functions of amphibian hepcidin have not been systematically investigated. The Chinese spiny frog (*Quasipaa spinosa*), distributed mainly in Southeast Asia, is an economically important aquaculture species whose production is frequently threatened by bacterial infections [26,27]. Yet, little is known about its innate immune effectors, and no *hepcidin* from this species has been functionally characterized.

In this study, we cloned and characterized a hepcidin homolog from the Chinese spiny frog, designated QsHep, and investigated its roles in antibacterial defense and immune regulation. We first analyzed the sequence features and tissue distribution of QsHep and examined its inducible expression following bacterial challenge. We then evaluated the antimicrobial spectrum and membrane-disrupting activity of synthetic QsHep, as well as its effects on chemotaxis, phagocytosis, and respiratory burst in primary macrophages. Finally, we assessed the in vivo protective efficacy of QsHep in an *Elizabethkingia miricola* infection model. By providing the first functional dissection of an amphibian hepcidin that integrates both antimicrobial and immunomodulatory readouts with survival outcomes in a natural host, this work expands our understanding of hepcidin biology in lower vertebrates and highlights QsHep as a promising candidate for peptide-based control of bacterial diseases in frog aquaculture.

## 2. Materials and Methods

### 2.1. Animals

The experimental subjects were sexually mature, post-metamorphic Chinese spiny frogs of both sexes, with a mean body weight of 150 ± 10 g, procured from a commercial farm in Lishui, China. Upon arrival, the frogs were inspected for general health; only those displaying normal posture and swimming behavior, normal feeding habits, and no visible skin lesions, ulcerations, or other external abnormalities were enrolled. The animals were acclimated for two weeks in a laboratory setting, during which they were monitored daily, and any individuals exhibiting lethargy, inappetence, or other overt signs of disease were excluded from subsequent experiments. The frogs were maintained in 100 L tanks and were fed a standard commercial diet twice a day. Water temperature (18–20 °C), pH (6.8–7.4), and dissolved oxygen (>6.0 mg/L) were monitored regularly, and water was partially renewed every 2–3 days to avoid the accumulation of nitrogenous wastes. The photoperiod was set at 12 h light: 12 h dark. These conditions were kept consistent across all tanks and experimental groups. All subsequent procedures performed in this research adhered strictly to the guidelines of China’s Experimental Animal Management Law and were approved by the Animal Research Ethics Committee of Lishui University.

### 2.2. Analysis of QsHep cDNA

The *QsHep* gene cDNA was derived from a liver transcriptome dataset (PRJNA1012438) that was generated and independently validated in our previous study, where sequencing depth and quality metrics were reported in detail [28]. The open reading frame (ORF) of *QsHep* was predicted using ORF Finder (https://www.ncbi.nlm.nih.gov/orffinder, accessed on 1 December 2025), and the cDNA sequence was confirmed by Sanger sequencing. The theoretical molecular mass and isoelectric point (*p*I) were calculated using ProtParam (https://web.expasy.org/protparam, accessed on 1 December 2025). A signal peptide was predicted with SignalP-5.0. Multiple sequence alignments were performed using the ClustalW algorithm, and phylogenetic analysis was conducted with MEGA X.

### 2.3. QsHep Expression Profiles

Four healthy adult Chinese spiny frogs that met the inclusion and health-screening criteria described in Section 2.1 were anesthetized and euthanized with MS-222 (400 ppm). Various tissues, including the liver, gut, muscle, skin, spleen, lung, heart, and kidney, were then collected and stored at −80 °C. To investigate the effect of bacterial challenge on *QsHep* expression, an *E. miricola* infection model was established as per a previous study [26]. Briefly, four healthy frogs were intraperitoneally injected with 100 μL of *E. miricola* (1.19 × 10^7^ CFU/mL), while a control group (*n* = 4) received an equal volume of 0.65% saline. All frogs were dissected at 12 h post-infection, and tissues including the liver, lung, kidney, and spleen were harvested and stored at −80 °C.

### 2.4. RT-qPCR

Total RNA was extracted from each sample using TRIzol reagent (Beyotime, Shanghai, China), followed by cDNA synthesis using a PrimeScript RT kit with gDNA Eraser (TaKaRa, Dalian, China). Subsequently, quantitative PCR was executed employing BeyoFast™ SYBR Green qPCR Mix (2X) (Beyotime). The cycling thresholds (Ct) of *QsHep* and *18S rRNA* were determined using a real-time PCR detection system (CFX96; Bio-Rad, Hercules, CA, USA). The primers used are listed in Table 1. The relative expression levels of *QsHep* and *18S rRNA* were computed utilizing the 2^−ΔΔCt^ method [29].

### 2.5. Antibacterial Assay

The mature form of the QsHep peptide (HSVLSICHYCCACCACCKNKGCGFCCMT) was chemically synthesized to a purity exceeding 95% (SynPeptide, Shanghai, China). The antibacterial activity of QsHep was evaluated against a broad spectrum of bacterial strains, including *Staphylococcus warneri*, *Shigella flexneri*, *Staphylococcus aureus*, *E. miricola*, *Aeromonas hydrophila*, and *Proteus mirabilis*. All bacterial strains were stored at −80 °C in broth containing 20% glycerol, then streaked onto appropriate agar plates and incubated overnight at 37 °C. A single colony of each strain was inoculated into Mueller–Hinton broth and cultured with shaking to the logarithmic growth phase. After logarithmic-phase culture, bacterial cells were harvested by centrifugation (5000× *g*, 5 min), washed twice with sterile phosphate-buffered saline (PBS, pH 7.4), and resuspended in PBS to a defined optical density so that all pellets were normalized to a comparable cell density before peptide treatment. The resulting suspensions were adjusted to a uniform moderate turbidity (approximately 10^8^ CFU/mL) and then diluted in broth to obtain a final inoculum of about 1 × 10^5^ CFU/mL in each well for the broth microdilution assay. Minimum inhibitory concentrations (MICs) were determined using a standard two-fold microdilution method [30]. Briefly, a geometric dilution series of QsHep (initial concentration 1 mg/mL, 1:2 serial dilutions) was prepared in sterile 96-well microplates; each well contained 10 μL peptide solution and 90 μL bacterial inoculum (1 × 10^5^ CFU/mL, log-phase). After 24 h incubation at the appropriate temperature for each strain, bacterial growth was quantified spectrophotometrically at 600 nm, and the MIC was defined as the lowest peptide concentration that completely inhibited visible growth.

### 2.6. Lactate Dehydrogenase Release Assay

A lactate dehydrogenase (LDH) release assay kit (Beyotime, Shanghai, China) was used to quantify the damage to bacterial cell membranes. Specifically, 1 × 10^9^ CFU of *E. miricola* was collected and resuspended PBS. Subsequently, the bacteria were exposed to QsHep (25, 50, or 100 μg/mL) and incubated at 37 °C for 2 h. Each well subsequently received a treatment of 60 μL of LDH assay working solution and was incubated at room temperature for 30 min, followed by absorbance measurement at 490 nm. Because LDH is a ubiquitous cytosolic enzyme in bacteria and the assay detects enzymatic activity rather than species-specific epitopes, we verified that detergent-lysed bacterial suspensions yielded strong LDH signals.

### 2.7. Cell Preparation

Macrophages were isolated from the spleens of healthy adult Chinese spiny frogs that satisfied the criteria described in Section 2.1, following modifications of a previously established protocol [31]. First, an undiluted Percoll separation solution was prepared by mixing 1.5 M NaCl with 100% Percoll at a ratio of 9:1. Then two gradient solutions were made: a 40% Percoll solution (6.14 mL of 0.15 M NaCl + 3.86 mL of 100% Percoll; buoyant density approximately 1.056 g/mL) and a 30% Percoll solution (7.14 mL of 0.15 M NaCl + 2.86 mL of 100% Percoll; buoyant density approximately 1.043 g/mL). The frogs were anesthetized by immersion in 0.6 g/L MS-222 and 0.6 g/L sodium bicarbonate, disinfected with 75% ethanol and euthanized while unconscious. Spleens were excised, washed three times with sterile PBS, and twice with serum-free medium. The spleens were then finely ground, passed through a 70 µm cell sieve, filtered and centrifuged at 1000 rpm for 5 min. The cell pellet was resuspended in the 40%/30% Percoll solutions, and after gradient centrifugation the cell layer above the 30% Percoll was collected. These cells were then resuspended in M199 medium containing 1000 U/mL penicillin, 1000 µg/mL streptomycin and 20% fetal bovine serum (FBS), and cultured at 25 °C under 2% CO_2_. Subsequently, the enriched macrophages were seeded at 5 × 10^6^ cells per plate and cultured for 12 h, after which non-adherent cells were removed. Cell viability and concentration were assessed using the trypan blue exclusion assay prior to use.

### 2.8. CCK-8 Assay

Cell viability was assessed using the CCK-8 kit (Elabscience Biotechnology, Wuhan, China). Briefly, approximately 5 × 10^3^ macrophages were inoculated into 96-well plates and treated with QsHep (0.1, 1.0, or 10.0 μg/mL) for 24 h. Bovine serum albumin (BSA) at 10.0 μg/mL served as a control. Subsequently, 10 μL of CCK-8 solution was added to each well and incubated for an additional 4 h. The absorbance of the reaction system was measured spectrophotometrically at 450 nm.

### 2.9. Chemotaxis Assay

Chemotaxis assays were conducted using a 24-well transwell chamber (Corning, Corning, NY, USA) following established methods [32]. The peptide QsHep was diluted in M199 to concentrations of 0, 0.1, 1.0, or 10.0 μg/mL, with 600 μL of each dilution added to the chamber, which was then covered with a 5 mm pore nitrocellulose filter membrane. Macrophages were added to the upper chamber and incubated for 24 h at 25 °C. Cells that migrated to the lower chamber were stained using the Wright-Giemsa stain and counted under 400× magnification, after which the migration ratio was calculated.

### 2.10. Phagocytosis Assay

Macrophages (5 × 10^5^) were seeded in a 6-well plate and incubated overnight. The following day, M199 with varying concentrations of QsHep (0, 0.1, 1.0, or 10.0 μg/mL) was added, and the cells were incubated for 12 h. Subsequently, the cells were harvested, re-suspended in 100 μL of FITC-dextran (1 mg/mL), and incubated for 30 min at 25 °C. Phagocytic activity was assessed using flow cytometry (Beckman CytoFLEX, Brea, CA, USA) [33].

### 2.11. Respiratory Burst Assay

The determination of the respiratory burst in macrophages was conducted in accordance with a previously described methodology [34]. Macrophages were pretreated with QsHep at varying concentrations (0, 0.1, 1.0, or 10.0 μg/mL) for 12 h. Following this, the cells were rinsed with sterile PBS and subjected to treatment with or without 0.1 μg/mL PMA. Each plate was treated with 1 mg/mL Nitroblue tetrazolium (NBT) and incubated at 24 °C for 1 h. The procedure was halted by the addition of 70% methanol, after which the cells were washed and dried. Formalin was dissolved in a solution of 120 µL 2 M KOH and 140 µL DMSO. An Ultrospec 1100 Pro UV/Vis spectrophotometer (Amersham Biosciences, Piscataway, NJ, USA) was used to measure the optical density at 620 nm.

### 2.12. Frog Survival Assays

Healthy adult frogs of both sexes that met the health-screening criteria described in Section 2.1 were randomly divided into three groups, each containing 16 individuals. Each frog was infected with 100 μL of *E. miricola* at an infectious dose of 1.19 × 10^6^ CFU. The experimental groups received an intraperitoneal injection of 0.1 μg/g or 1.0 μg/g of QsHep 30 min post-infection, while the control group was injected with 1.0 μg/g BSA. Survival times were monitored for 7 d, and the frogs’ health was assessed every 12 h. Surviving frogs were euthanized at the end of the observation period.

### 2.13. Statistical Analyses

All data are expressed as means ± SEM. For each experiment, n denotes the number of biological replicates (independent cell preparations or individual frogs), and the exact n for each assay is indicated in the corresponding figure legends. For comparisons involving more than two groups within a single factor (different concentrations of QsHep plus a common control under the same condition), data were analyzed by one-way analysis of variance (ANOVA) followed by Tukey’s multiple-comparison test. When only two groups were compared, an unpaired Student’s *t*-test was used. For the respiratory burst assay, our primary comparisons were QsHep versus BSA in unstimulated cells and QsHep + PMA versus BSA + PMA in PMA-stimulated cells; therefore, these data were analyzed using one-way ANOVA within each stimulation condition rather than a full two-way ANOVA. Statistical analyses were performed using SPSS software (version 13.0, SPSS Inc., Chicago, IL, USA), and differences were considered statistically significant at *p* < 0.05.

## 3. Results

### 3.1. Characterization of QsHep

**Figure 1:**
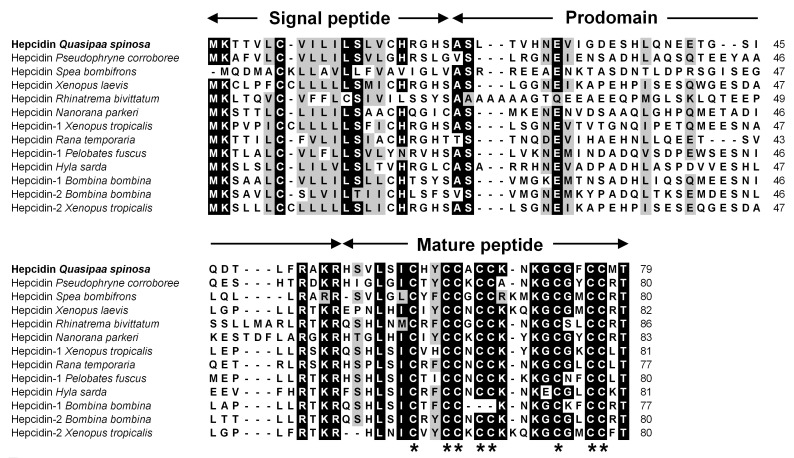
Sequence alignment of amphibian hepcidin homologs. A shading threshold of 70% was applied, wherein similar residues are denoted in gray, identical residues in black, and alignment gaps are represented by hyphens (-). The conserved eight cysteine residues are marked with “*”. The numerals on the right side correspond to the amino acid number.

The *QsHep* cDNA was identified from the liver transcriptome and submitted to GenBank (accession number: PQ117753). It features a 240-nucleotide ORF that encodes a 79 aa polypeptide. This peptide consists of a signal peptide, prodomain, and mature peptide, which is predicted to have a molecular weight of 2.72 kDa and a *p*I of 8.21. Comparative analysis of the amino acid sequences of QsHep and other amphibian hepcidins indicated substantial divergence in the signal peptide and prodomain regions, whereas the functional mature peptide exhibited a higher degree of conservation. Notably, the mature amphibian hepcidin peptide contained eight conserved cysteine residues (Figure 1).

**Figure 2:**
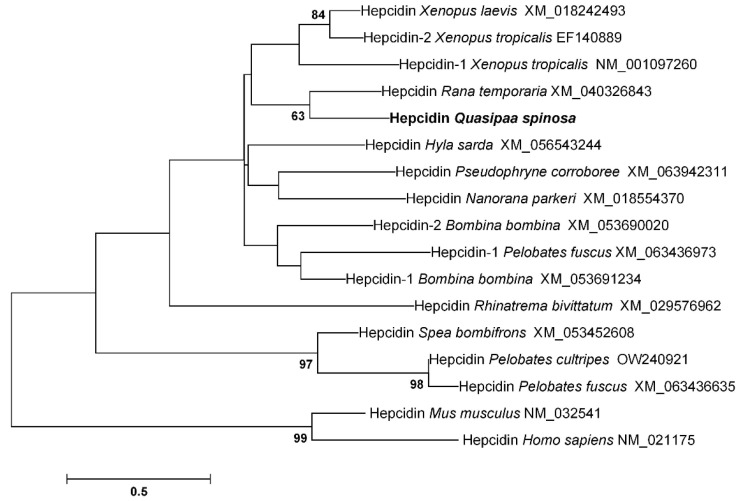
Phylogenetic analysis of QsHep. Branching point values show the percentage of trees with that grouping, based on 1000 bootstrap replicates (displayed only if over 60%). The scale bar indicates substitutions per base.

Phylogenetic tree analysis demonstrated that QsHep exhibited the highest degree of similarity to hepcidin of *Rana temporaria* (Figure 2).

### 3.2. The Expression of QsHep

**Figure 3:**
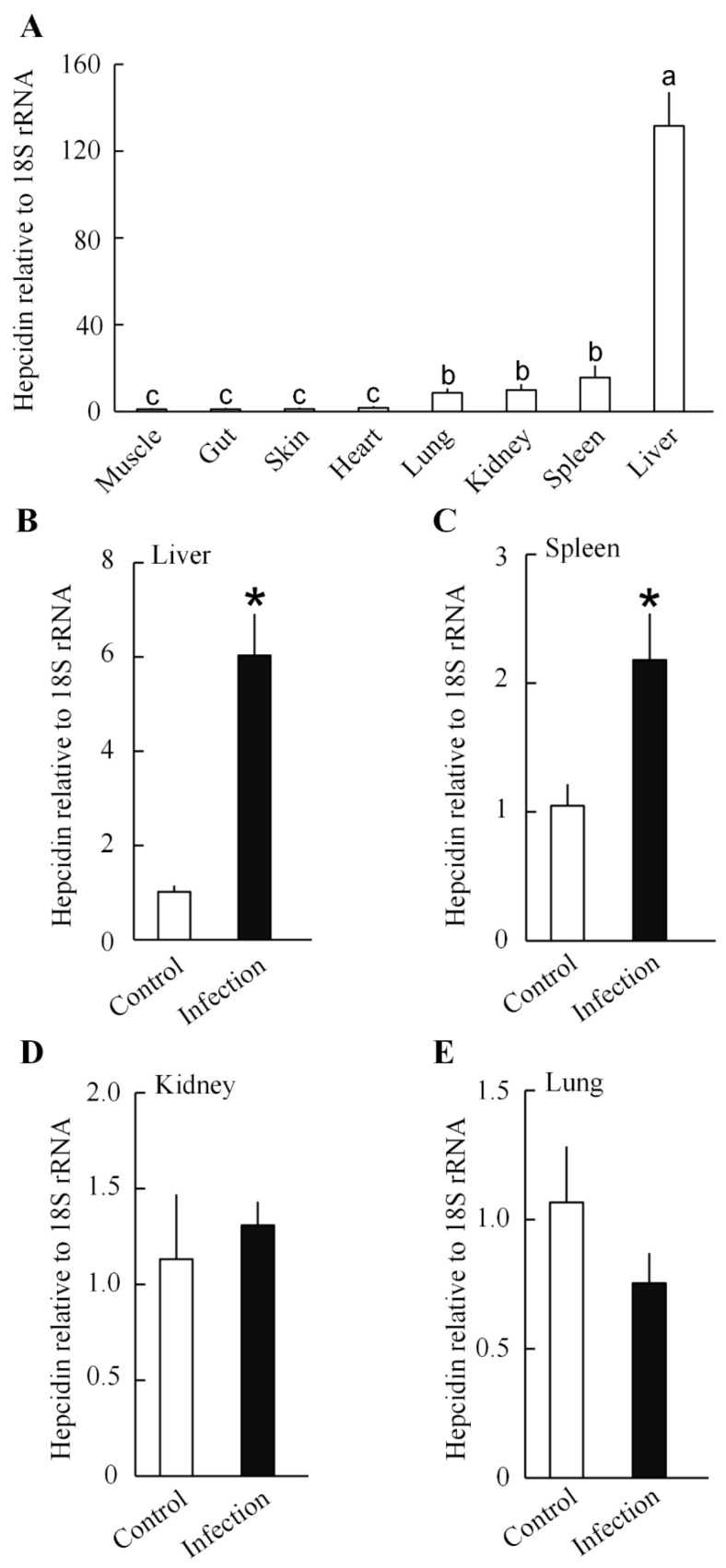
qPCR analysis of *QsHep* expression in Chinese spiny frog tissues. (**A**) The tissue-specific expression of the *QsHep* gene. Means with diferent letters differ signifcantly (*p* < 0.05). (**B**–**E**) Changes in the expression of the *QsHep* gene were observed after infection with *Elizabethkingia miricola*. Data are expressed as mean ± SEM; *n* = 4; * *p* < 0.05.

All tested tissues expressed *QsHep*, with the liver expressing the highest levels (Figure 3A). *E. miricola* infection significantly increased *QsHep* expression in the liver (5.93-fold) and spleen (2.08-fold) (Figure 3B,C), but not in the kidneys or lungs (Figure 3D,E).

Means with diferent letters differ signifcantly (Tukey’s post hoc test, *p* < 0.05).

### 3.3. QsHep In Vitro Antibacterial Activity

QsHep showed varying antibacterial effects, most effectively against *S. warneri* (MIC = 3.125 μg/mL). It had MIC values of 25 μg/mL for *S. flexneri* and *E. miricola*, and 50 μg/mL for *S. aureus*. However, QsHep was not significantly effective against *P. mirabilis* or *A. hydrophila* (Table 2).

### 3.4. Effects of QsHep on Elizabethkingia miricola Cell Membrane Integrity

**Figure 4:**
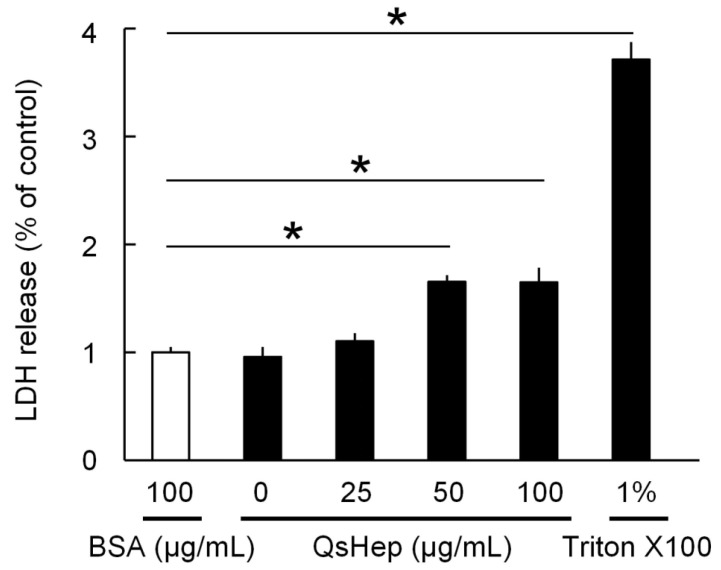
Effect of QsHep on the integrity of the *Elizabethkingia miricola* cell membrane. LDH release was calculated as the fold-change relative to the baseline value of 1 using BSA as a negative control. Data are expressed as mean ± SEM; *n* = 4; * *p* < 0.05.

The LDH assay showed that QsHep damaged the cell membrane of *E. miricola*, with the greatest damage at 100 μg/mL, causing a 1.65-fold increase in LDH release compared to the control. No significant effects were observed at 25 μg/mL (Figure 4).

### 3.5. Effect of QsHep on Macrophage Viability and Chemotaxis

**Figure 5:**
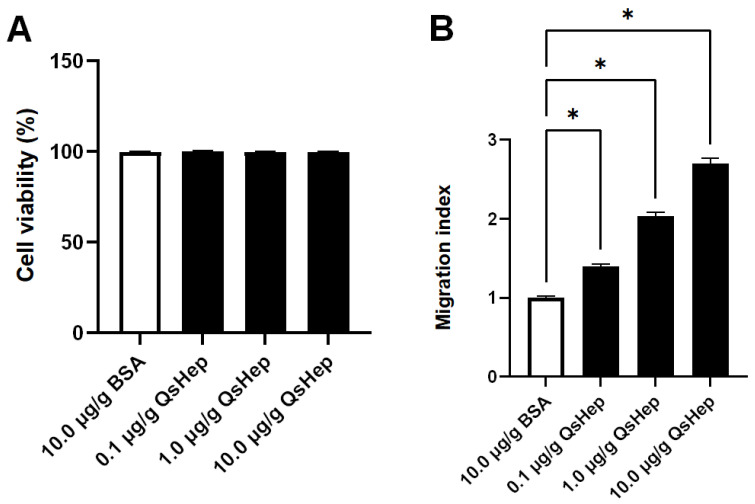
Effects of QsHep on macrophage viability and chemotaxis. (**A**) Cell viability after treatment with different concentrations of QsHep (0.1, 1.0, and 10.0 μg/mL) for 24 h, as assessed by CCK-8 assay. (**B**) Chemotactic response of macrophages to QsHep, quantified using Transwell chambers after 24 h of incubation. BSA was used as a negative control in both assays. Data are expressed as the mean ± SEM of four (viability) or three (chemotaxis) independent experiments. Statistical significance versus the BSA control was determined by one-way ANOVA (* *p* < 0.05).

The CCK-8 assay showed that treatment with QsHep at 0.1, 1.0, and 10.0 μg/mL did not affect cell viability, indicating that QsHep exhibits no obvious cytotoxicity toward Chinese spiny frog cells within this concentration range and has good in vitro safety (Figure 5A).

Macrophages showed an effective chemotactic response to QsHep at 0.1, 1.0, and 10.0 μg/mL. Cell migration increased 2.71 times in the 10.0 μg/mL QsHep group compared to the BSA group (Figure 5B).

### 3.6. Effect of QsHep on the Phagocytosis of Macrophages

**Figure 6:**
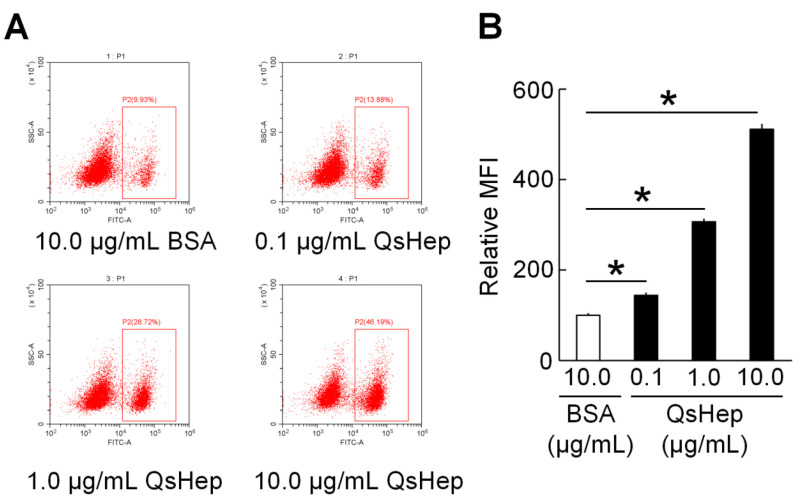
The effect of QsHep on macrophage phagocytosis. (**A**) Representative flow-cytometry dot plots of FITC-dextran uptake in macrophages treated with 10.0 µg/mL BSA (control) or QsHep at 0.1, 1.0, and 10.0 µg/mL. The gated region (P2) indicates FITC-positive cells. (**B**) Quantification of relative mean fluorescence intensity (MFI) of FITC-dextran. The MFI was expressed as a fold change relative to the group treated with BSA, which was assigned a value of 100. Data are expressed as mean ± SEM; *n* = 4; * *p* < 0.05.

Phagocytosis of FITC-dextran was significantly augmented by QsHep treatment in macrophages. Notably, at a concentration of 10.0 µg/mL, phagocytosis was observed to be 5.11 times greater than that of the BSA control. Furthermore, the higher the concentration of QsHep, the more pronounced the phagocytic activity (Figure 6).

### 3.7. Effect of QsHep on Macrophage Respiratory Burst

**Figure 7:**
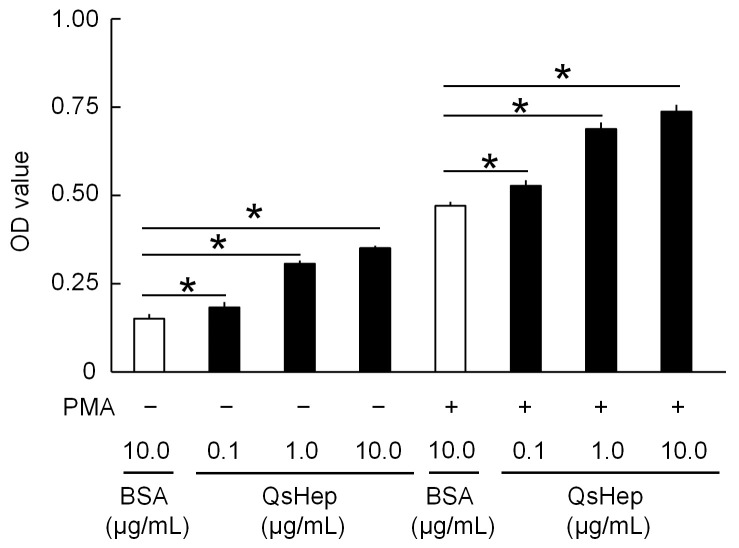
Effect of QsHep on the respiratory burst of macrophages. Data are expressed as means ± SEM. *n* = 4. * *p* < 0.05.

The respiratory burst of macrophages was analyzed following QsHep stimulation. It was observed that QsHep induced a respiratory burst, with the highest induction occurring at 10.0 μg/mL. This concentration resulted in a 2.32-fold increase compared to the BSA control group. Additionally, co-stimulation with PMA and QsHep significantly enhanced the respiratory burst of macrophages, particularly at the 10.0 μg/mL QsHep concentration in conjunction with PMA, which showed a 1.57-fold increase relative to the PMA and BSA co-treatment (Figure 7).

### 3.8. Effect of the QsHep on Frog Survival After Elizabethkingia miricola Challenge

**Figure 8:**
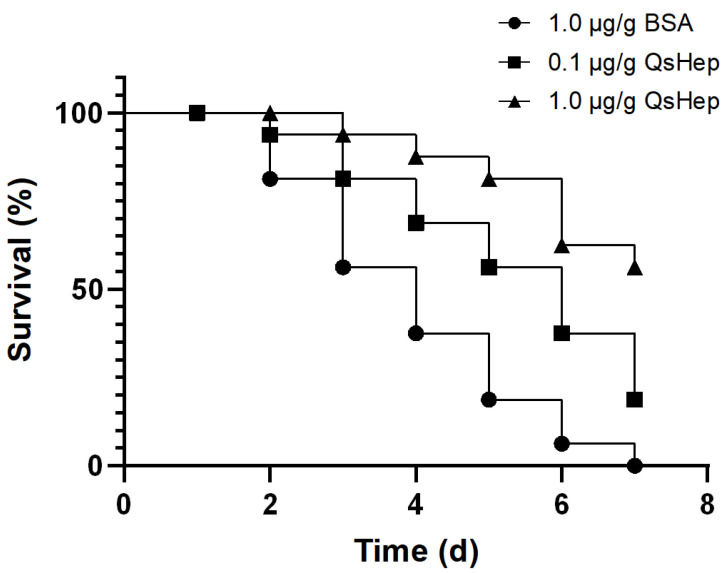
The effect of QsHep on survival rate after *Elizabethkingia miricola* infection. The frogs in the experimental groups received 0.1 μg/g or 1.0 μg/g QsHep (ip) 30 min after *E. miricola* infection. Frogs were monitored for signs of sickness and mortality every 12 h for 7 d. There were 16 frogs in each group.

In the BSA group, mortality progressed rapidly: survival dropped to 56.25% by day 3 and all frogs had died by day 7 (0/16, 0%). In contrast, QsHep treatment markedly improved survival in a dose-dependent manner. In the 0.1 μg/g group, 81.25% of frogs remained alive at day 3 and 18.75% (3/16) were still alive at day 7. The 1.0 μg/g group showed the strongest protection, with 93.75% survival at day 3 and 56.25% (9/16) of frogs surviving to day 7. Overall, QsHep significantly prolonged survival of *E. miricola*-infected Chinese spiny frogs, and the high dose (1.0 μg/g) provided greater protection than the low dose (0.1 μg/g) (Figure 8).

## 4. Discussion

This study focused on the *hepcidin* gene in amphibians, particularly in the Chinese spiny frog. The identified amino acid sequence, QsHep, comprises a signal peptide, a prodomain, and a mature peptide. The mature peptide contains eight conserved cysteine residues that form four disulfide bonds, which are crucial for proper folding [18] and antimicrobial activity [35]. The *QsHep* gene was expressed in multiple healthy tissues, with the liver showing the highest transcript levels, in agreement with previous observations for hepcidin in *X. tropicalis* [25]. Moreover, *QsHep* expression in the liver and spleen was significantly upregulated after *A. hydrophila* infection, consistent with reports in other vertebrates in which hepcidin is rapidly induced in response to bacterial challenge [14,36,37]. For example, in *Hemibarbus labeo*, *hepcidin* expression is markedly increased in the liver, spleen and head kidney following *A. hydrophila* infection [14]. Together, these data support a conserved role for hepcidin as a key component of the amphibian innate immune response.

This work also represents, to our knowledge, the first characterization of the antimicrobial properties of an amphibian hepcidin peptide. Chemically synthesized QsHep showed clear antibacterial activity against *S. warneri*, *S. flexneri*, *S. aureus* and *E. miricola*. Hepcidins from mammals, fish and reptiles have likewise been reported to possess broad-spectrum antimicrobial activity [16,17,19]; for instance, recombinant hepcidin from *Crocodylus siamensis* strongly inhibits the growth of *Escherichia coli*, *Aeromonas sobria*, *Staphylococcus aureus* and *Bacillus subtilis* in vitro [17]. In our LDH-release assay, QsHep significantly increased LDH release from *E. miricola*, indicating disruption of bacterial membrane integrity. This observation is consistent with previous studies showing that hepcidins damage bacterial cell membranes, as visualized by electron microscopy [16,38]. Such membrane disruption leads to leakage of intracellular contents and ultimately bacterial death [39,40].

Beyond its direct bactericidal effects, QsHep also exhibited immunomodulatory activities. QsHep promoted the chemotaxis of primary cultured macrophages, resembling the activity of chemokines and suggesting a role in recruiting phagocytes to sites of infection. In addition, QsHep significantly enhanced the phagocytic activity of these primary macrophages, in line with studies showing that fish hepcidins can boost phagocytosis by leukocytes and macrophages [36,41]. QsHep also augmented the respiratory burst in primary macrophages. Because phagocyte-mediated killing of bacteria relies on the generation of reactive oxygen and nitrogen species, this enhanced respiratory burst indicates that QsHep can potentiate macrophage microbicidal functions [42].

Consistent with its in vitro antimicrobial and immunomodulatory activities, QsHep also conferred clear protection in the in vivo challenge model. Following *E. miricola* infection, all frogs in the BSA control group succumbed by day 7, whereas QsHep administration significantly improved survival in a dose-dependent manner, with the 1.0 μg/g group retaining more than twice as many survivors as the 0.1 μg/g group at the end of the experiment (Figure 8). This pattern suggests that QsHep can effectively limit systemic *E. miricola* infection when present at sufficient concentrations, likely through a combination of direct bacterial killing and enhancement of macrophage chemotaxis, phagocytosis and respiratory burst. Similar in vivo protective effects have been reported for hepcidin or hepcidin-derived peptides in other vertebrates, where synthetic or recombinant hepcidins administered by injection or diet significantly reduced mortality, increased relative percent survival and/or lowered pathogen loads after bacterial or viral challenge in teleost fish and mammals [43,44,45,46]. These convergent data support a conserved role of hepcidin peptides as effector molecules that not only modulate iron homeostasis and innate immune functions but also improve host survival during systemic infection.

From an applied perspective, the dual antimicrobial and immunomodulatory properties of QsHep suggest that this peptide, or QsHep-derived analogs, could be exploited in prophylactic strategies for disease control in aquaculture [43,47,48,49]. In teleosts, several studies have shown that hepcidin peptides can be delivered as feed additives or oral supplements to enhance basal immunity, modulate gut microbiota, improve growth performance, and increase resistance to bacterial challenge [43,47,48]. For example, long-term dietary supplementation with recombinant tilapia hepcidin 2-3 improved immune-related gene expression, reshaped the gut microbiota, and enhanced survival of grouper after *Vibrio* challenge [47], whereas oral administration of grass carp hepcidin, alone or in combination with chitosan, benefited growth, innate immune parameters, and resistance to bacterial infection in grass carp [43,48]. Recent reviews on fish-derived hepcidins and other antimicrobial peptides further emphasize their potential as prophylactic immunostimulants and alternatives to antibiotics in aquaculture [43,49].

By analogy, prophylactic administration of QsHep in Chinese spiny frog culture—for example, via in-feed supplementation, immersion during high-risk periods (such as transport, overwintering, or crowding stress), or as an adjunct to vaccination—may help to prime innate defenses and reduce the incidence of opportunistic infections. Amphibian skin and liver-derived host-defense peptides, including hepcidins and frog skin antimicrobial peptides, are increasingly recognized as promising templates for anti-infective development because of their broad-spectrum activity and relatively low propensity to select for resistance [50]. Future work should therefore evaluate prophylactic QsHep regimens in vivo (dose, route, timing) and assess long-term impacts on microbiota, iron homeostasis, and host fitness, in order to determine whether QsHep-based interventions can be safely integrated into sustainable frog-farming practices.

## 5. Conclusions

In summary, we identified and characterized a *hepcidin* gene from the Chinese spiny frog (*QsHep*) that shows typical hepcidin features and is strongly induced after bacterial challenge. Synthesized QsHep displayed broad-spectrum antibacterial activity by damaging bacterial membranes, promoted chemotaxis, phagocytosis and respiratory burst in primary macrophages without obvious cytotoxicity, and significantly improved the survival of *E. miricola*-infected frogs in vivo. These findings indicate that amphibian hepcidin functions as both an antimicrobial effector and an immunomodulatory peptide, and highlight QsHep as a promising candidate for controlling bacterial diseases in aquaculture.

## Figures and Tables

**Table 1:** Primer sequences.

Gene	Primer	Sequence (5′–3′)
*Hepcidin*	*Hep*-t(+)	ATCCTCATCCTCTCCCTGGT
	*Hep*-t(−)	GATGGAAAGGACGGAATGGC
*18S rRNA*	*18S rRNA*-t(+)	TTAGAGGGACAAGTGGCGTT
	*18S rRNA*-t(−)	TGCAATCCCCGATCCCTATC

**Table 2:** Antibacterial activity of synthetic QsHep peptide.

Bacteria	Isolate/Strain	MIC (μg/mL)
*Staphylococcus warneri*	ATCC49454	3.125
*Shigella flexneri*	ATCC12022	25
*Staphylococcus aureus*	ATCC6538	50
*Elizabethkingia miricola*	ATCC33958	25
*Aeromonas hydrophila*	ATCC7966	-
*Proteus mirabilis*	ATCC25933	-

-: no inhibition detected at 100 μg/mL.

## Data Availability

The data that support the findings of this study are available on request from the corresponding author. The data are not publicly available due to privacy or ethical restrictions.
